# Molecular Cloning and Functional Expression of a Δ9- Fatty Acid Desaturase from an Antarctic *Pseudomonas* sp. A3

**DOI:** 10.1371/journal.pone.0160681

**Published:** 2016-08-05

**Authors:** Lawal Garba, Mohd Shukuri Mohamad Ali, Siti Nurbaya Oslan, Raja Noor Zaliha Raja Abd Rahman

**Affiliations:** 1 Enzyme and Microbial Technology Research Centre, Faculty of Biotechnology and Biomolecular Sciences, University Putra Malaysia, 43400 UPM Serdang, Selangor Darul Ehsan, Malaysia; 2 Department of Microbiology, Faculty of Science, Gombe State University, Tudun Wada Gombe, P.M.B 127, Gombe State, Nigeria; Louisiana State University Health Sciences Center, UNITED STATES

## Abstract

Fatty acid desaturase enzymes play an essential role in the synthesis of unsaturated fatty acids. *Pseudomonas sp*. A3 was found to produce a large amount of palmitoleic and oleic acids after incubation at low temperatures. Using polymerase Chain Reaction (PCR), a novel Δ9- fatty acid desaturase gene was isolated, cloned, and successfully expressed in *Escherichia coli*. The gene was designated as *PA3FAD9* and has an open reading frame of 1,185 bp which codes for 394 amino acids with a predicted molecular weight of 45 kDa. The activity of the gene product was confirmed via GCMS, which showed a functional putative Δ9-fatty acid desaturase capable of increasing the total amount of cellular unsaturated fatty acids of the *E*. *coli* cells expressing the gene. The results demonstrate that the cellular palmitoleic acids have increased two-fold upon expression at 15°C using only 0.1 mM IPTG. Therefore, *PA3FAD9* from *Pseudomonas sp*.A3 codes for a Δ9-fatty acid desaturase-like protein which was actively expressed in *E*. *coli*.

## Introduction

Fatty acid desaturase enzymes catalyse the desaturation reactions that introduce double bonds into fatty acyl chains. The enzymes are broadly divided into two phylogenetically unrelated groups of soluble acyl-acyl carrier protein desaturases and a more widespread group of membrane-bound desaturases, which are made up of acyl-lipid and acyl-coA desaturases [1,2]. Desaturase enzymes have been reported in all members of prokaryotes and eukaryotic organisms [3,2]. Although there are differences in substrate specificity and cellular location, desaturase enzymes utilise the same reaction mechanisms, which require an electron transport system of either ferredoxin-NADP+ oxidoreductase and ferredoxin, or cytochrome b5 reductase, cytochrome b5, NAD (P)H, and molecular oxygen [3,4].

The Δ9-desaturase enzymes insert the first double bond into saturated fatty acyl chains, initiating the first step of polyunsaturated fatty acids (PUFAs) production, as most other desaturases and elongases require an existing double bond at the C-9 position for further desaturation [5, 3]. Some PUFAs like ω-3 fatty acids offer several benefits to the body, such as promoting good health by reducing cholesterols levels and alleviating the prevalence of many fatal diseases such as stroke, hypertension, diabetes, depression, asthma, osteoporosis, schizophrenia, colon cancer, rheumatoid arthritis, and prostate cancer [6]. However, it is generally believed that higher organisms are unable to produce ω-3 Fatty acids *de novo* such that they must be obtained from diets. Most of the currently available ω-3 fatty acids are sourced from fish species such as salmon, mackerel, sardine, and herring [7–9]. The use of essential fatty acids obtained from fish may be associated with the disadvantages of contamination by environmental pollution and an undesirable fishy smell or taste. Moreover, fatty acids that are sourced from marine fish may be a complex mixture of varied chain lengths and degrees of unsaturation requiring expensive purification procedures. In order to improve on fish fatty acids, various alternative production sources such as Antarctic bacteria are being considered. Having understood the significance of Δ9-fatty acid desaturases in unsaturated fatty acid production, the coding genes for these enzymes have been cloned and expressed from both prokaryotes and eukaryotic organisms [1, 3–4, 10].

Although many genes for Δ9-desaturases have been reported across living kingdoms including plants, mammals, fungi and some bacteria [11–12], a large number of open reading frames (ORFs) with significant similarity to Δ9- fatty acid desaturases have been sequenced in the genomes of several *Pseudomonas species* and submitted to the public database. However, most of these ORFs have yet to be functionally demonstrated [13]. In this report, we have successfully cloned and expressed an active Δ9- fatty acid desaturase from an Antarctic bacterium, *Pseudomonas sp*. A3. The enzyme may be used to enhance polyunsaturated fatty acids production, particularly in the event of coexpression with genes that code for other desaturase and elongase enzymes in an appropriate host.

## Results

### Fatty acids profile of wild-type *Pseudomonas sp*., A3 in relation to temperature changes

**Fig 1:**
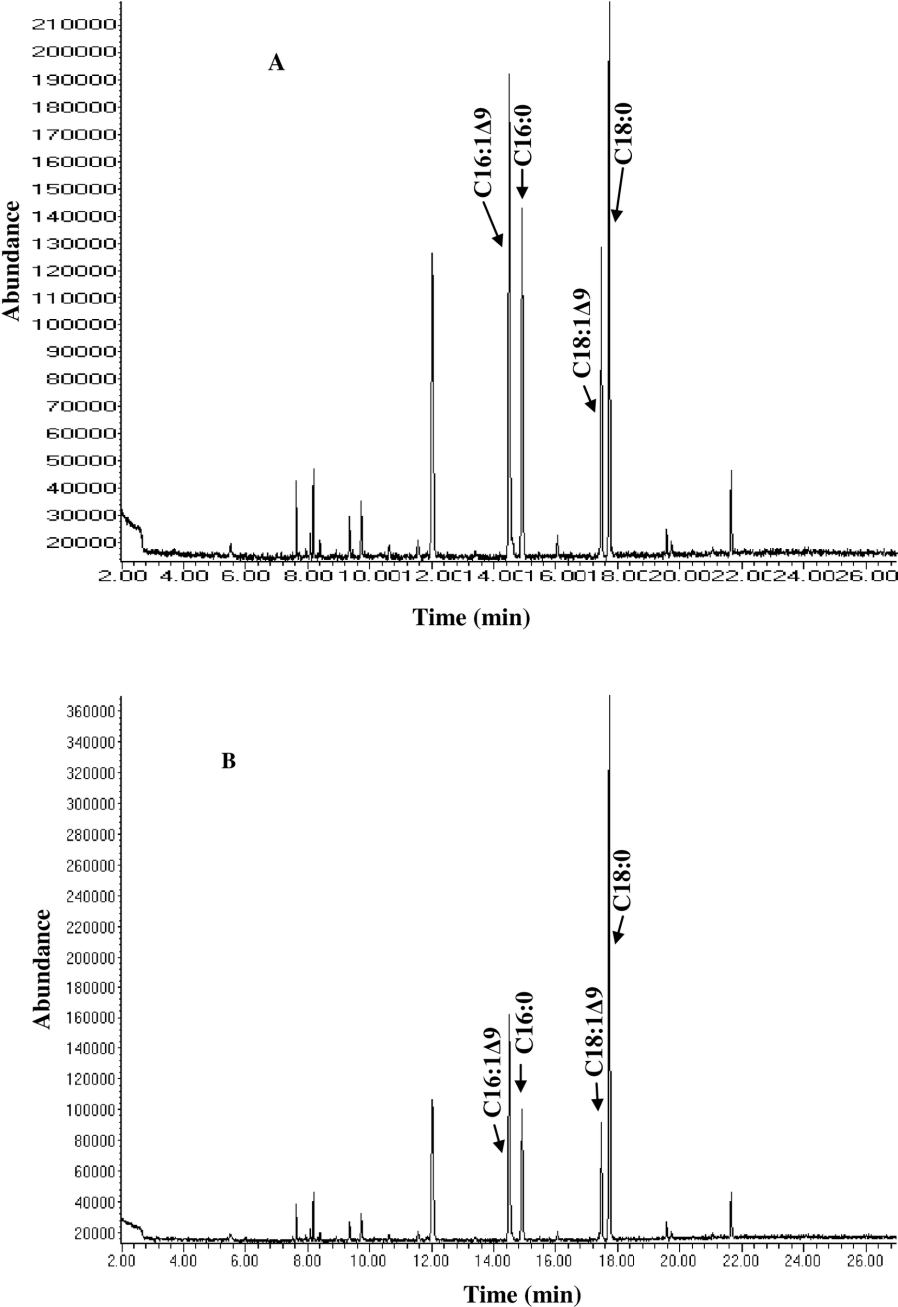
**Gas chromatograms of fatty acids analysed from *Pseudomonas sp*., A3 cultured at 4°C, 96 h (A) or 15°C, 48 h and 4°C, 48 h (B).** The arrowheads indicate the C16 and C18 fatty acids with the positions of double bonds in palmitoleic (C16:1Δ9) and oleic acids (C18:1Δ9) identified according to MS data.

The previous study has confirmed the ability of *Pseudomonas sp*., A3 to produce monounsaturated fatty acids at low temperature [14]. To investigate the production of unsaturated fatty acids in response to temperature changes, the bacterium was cultured at different incubation conditions and analysed its fatty acids using GCMS (Table 1). According to the analysis, palmitoleic (C16:1Δ9) and oleic acids (C18:1Δ9) were the only unsaturated fatty acids identified (Fig 1), cumulatively accounting for a total unsaturation of 63.71%, 51.05%, 23.34% and 27.7% at 4, (15+4), (4+15) and 15°C respectively (Table 1). The findings are in line with previous reports that psychrophilic organisms increase the amount of their cellular unsaturated fatty acids in response to incremental decreases in growth temperatures [12], suggesting that *Pseudomonas sp*. A3 as a potential Δ9-fatty acid desaturase producer.

**Table 1:** Overall fatty acids composition of *Pseudomonas sp*., A3 at different temperatures.

Incubation conditions	Fatty acid (%)								Unsaturation (%)
	C8:0	C10:0	C12:0	C14:0	C16:1Δ9	C16:0	C18:1Δ9	C18:0	
4°C,96h	1.10 ±0.71	0.37 ±0.32	2.49 ±0.32	1.38 ±0.37	47.41 ±2.17	39.80 ±5.08	16.30 ±3.11	5.22 ±0.47	63.71
15+4°C,96h	2.02 ±0.16	2.50 ±0.33	1.25 ±0.11	1.38 ±0.37	36.88 ±12.50	27.10 ±2.43	14.17 ±2.71	25.26 ±4.05	51.05
4+15°C,96h	0.31 ±0.07	0.04 ±0.01	0.46 ±0.29	0.74 ±0.18	15.30 ±0.97	39.04 ±22.65	8.04 ±1.01	6.56 ±0.83	23.34
15°C,96h	3.45 ±0.83	5.27 ±0.10	6.80 ±0.71	9.99 ±2.08	19.20 ±0.83	64.48 ±2.94	8.50 ±1.22	35.39 ±4.01	27.7

The overall fatty acids composition of *Pseudomonas sp*. A3 analysed using GCMS. Both saturated and unsaturated fatty acids of different chain lengths ranging from C8 to C18 were identified. Palmitoleic (C16:1Δ9) and oleic acids (C18:1Δ9) were the only unsaturated fatty acids observed based on the MS data. Results are means ± S.D of three independent experiments.

### Cloning of *PA3FAD9* gene

**Fig 2:**
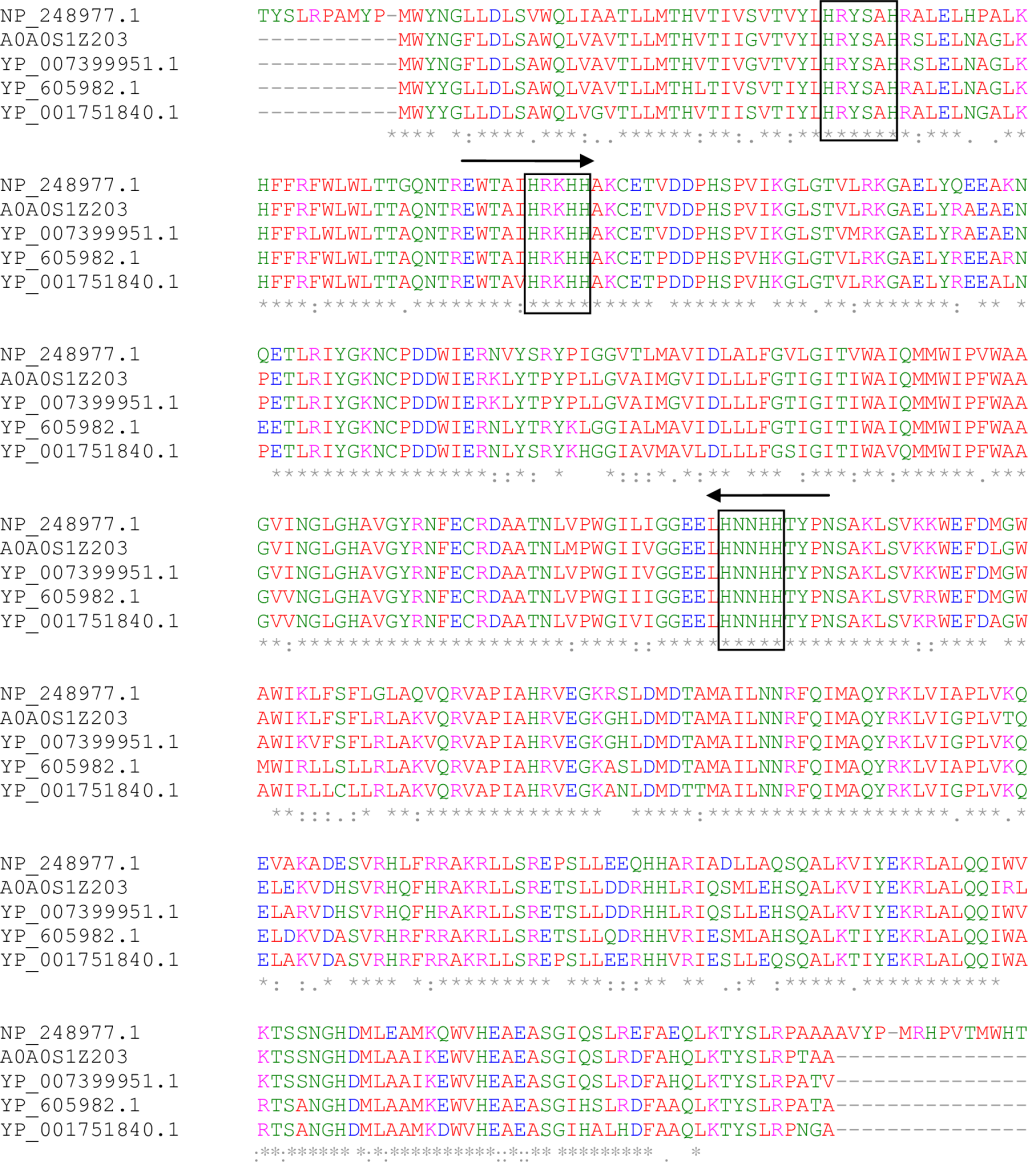
Multiple sequences alignments of fatty acid desaturase in *Pseudomonas sp*., A3 (GenBank accession no: A0A0S1Z203) and other *Pseudomonas species* including *Pseudomonas entomophila* l48 (GenBank accession no: YP_605982.1), *Pseudomonas aeruginosa* PAO1 (GenBank accession no: NP_248977.1), *Pseudomonas poae* (GenBank accession no: YP_007399951.1) and *Pseudomonas putida* w619 (GenBank accession no: YP_001751840.1) performed using Clustal 2.0[16]. Three histidine consensus regions (HXXXXH and two HXXHH) are shown in circled boxes, while the arrowheads show the sequences used in the degenerate primers design.

**Fig 3:**
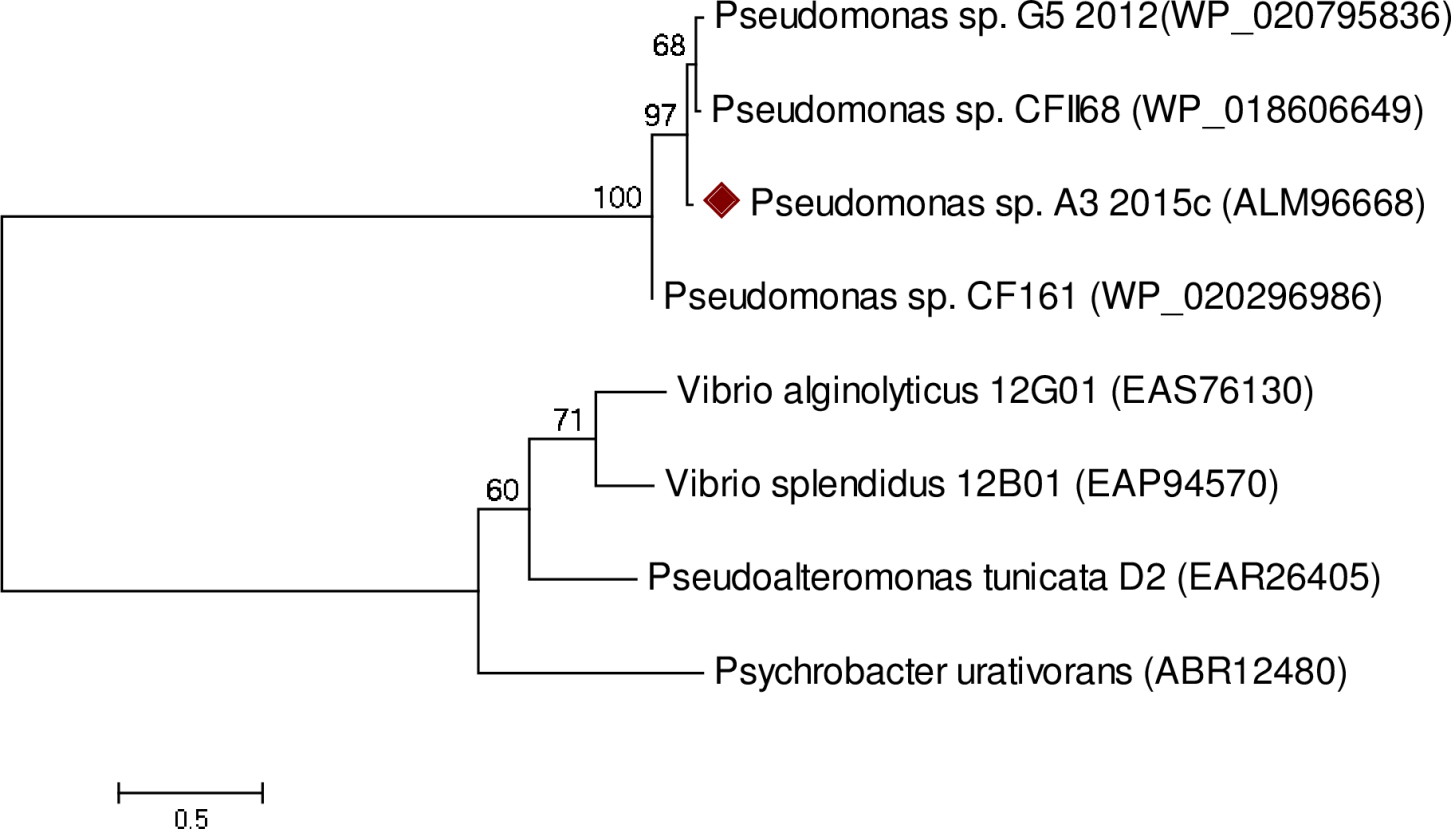
Phylogenetic analysis of *Pseudomonas sp*., A3 desaturase sequence (ALM96668), sequences in other *Pseudomonas species* and some bacteria. The numbers in parentheses show the GenBank accession numbers. The sequences alignments and construction of phylogenetic tree were performed using a neighbour-joining tree in MEGA 6.0 [17] using default parameters.

A gene fragment of about 400 bp was isolated from *Pseudomonas sp*., A3 by PCR using degenerate primers derived from two sequences of RKHHAKCE and HNNHHTYP for forward and reverse primers, respectively, corresponding to the highly conserved histidine regions of putative fatty acid desaturases in *Pseudomonas species* as shown in Fig 2. The gene fragment demonstrated a high degree of similarity to putative desaturase genes in other *Pseudomonas species*. Isolation of 1,200 bp as the expected size of the gene was achieved via PCR (S1 Fig). The gene information was confirmed by sequencing and submitting to GenBank (accession no. KT033499). It was designated as PA3FAD9, with an open reading frame of 1,185 bp, coding for 394 amino acid polypeptides of a molecular weight of 45 kDa and a theoretical isoelectric point of 9.48. The histidine-rich boxes which are found in all insoluble membrane-bound desaturases (acyl-lipid and acyl-coA desaturases) [1, 15] were observed as HxxxxH (34–39), HxxHH (71–75) and HxxHH (206–110). The role of the three histidine-rich regions was investigated in some desaturase enzymes including Stearoyl-CoA desaturase and Δ12 acyl-lipid desaturase using site-directed mutagenesis [1, 15]. They are potential ligands for iron atoms and are considered to be the main sites for the enzymes catalytic activity. Analysis of the phylogenetic tree has revealed significant similarities among *Pseudomonas sp*., A3 fatty acid desaturase, and putative desaturases in certain related species (Fig 3).

### Analysis of Amino Acid Sequences

The primary structure of the putative desaturase from *Pseudomonas sp*., A3 has revealed significant similarity to those fatty acid desaturase sequences deposited in the GenBank based on the Blast search showing 98% identity to *Pseudomonas fluorescens* WH6, 94% identity to *Pseudomonas sp*. CF161, 93% identity to *Pseudomonas sp*. G5 (2012), 93% identity to *Pseudomonas sp*. OS17, 93% identity to *Pseudomonas sp*. UW4 and 92% identity to *Pseudomonas sp*. CF1168. Although membrane-bound desaturase genes sequences from different *Pseudomonas species* have been deposited in the GenBank, to the best of our knowledge the gene reported in this study is the first to be functionally expressed.

**Fig 4:**
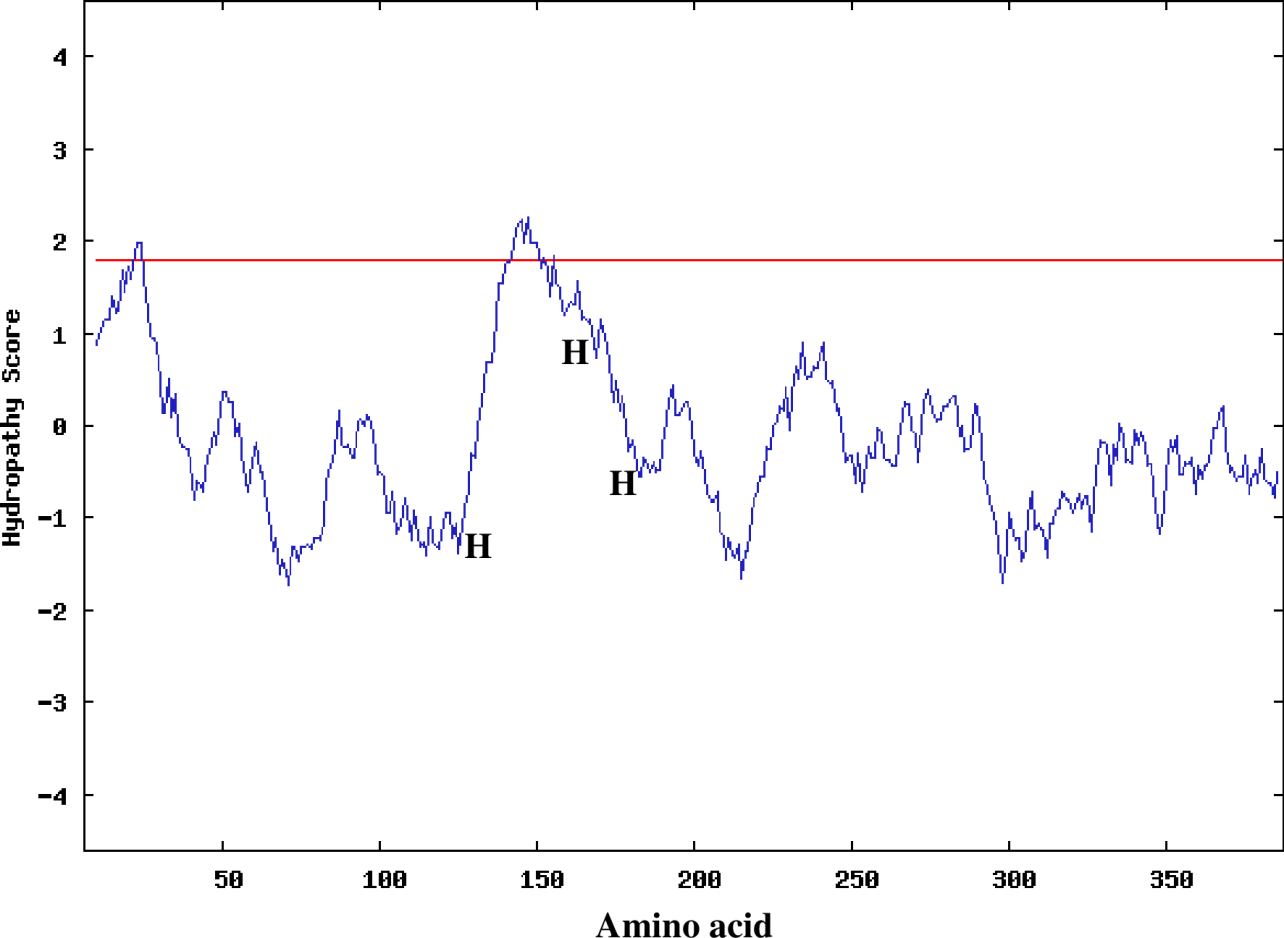
Kyte-Doolittle Hydropathy analysis of the amino acids sequence of *Pseudomonas sp*., A3 desaturase. The search for a transmembrane region in this protein was carried out using a window size of 19 amino acids. Peaks above the red line show the possible transmembrane regions, whereas the histidine clusters of putative *Pseudomonas sp*., A3 are shown by H’s.

The membrane-bound desaturases of *Mortorella alpine* have been successfully expressed and purified [10]. However, to date, there is no report on the three-dimensional structure of these recalcitrant to purify proteins. The proposed topology model of membrane enzymes that could be applied to many membrane desaturases was reported from stearoyl-CoA desaturase in mouse [1, 18], membrane-bound alkaline hydroxylase in *Pseudomonas oleovorans* [1, 19] and acyl-lipid Δ5 desaturase in *Bacillus subtilis* [1, 20]. Distribution of hydrophobic amino acids of *Pseudomonas sp*., A3 desaturase that is unique to membrane proteins was investigated using the Kyte-Doolittle hydropathy window scale 19 (http://www.expasy.ch/cgi-bin/protscale.p1) [21] (Fig 4). According to TM helices prediction, the protein has two hydrophobic domains located between amino acids 10–32 and 135–157 and thought to be long enough to span the membrane bilayer two times with the two protein termini facing the cytosol. This prediction is in line with the earlier report on the topology models of Δ9-desaturases [3, 18, 22]. The amino acids ‘‘MWYNGFLDLSAWQLVAVTLLMT” were predicted as the potential N-terminal signal peptide based on the SignalP-NN prediction [3, 23, 24].

### Heterologous Expression of *PA3FAD9* and *in vivo* analysis of Δ9- fatty acid desaturase-like protein

The expression of PA3FAD9 gene was studied in *E*. *coli* as described in the methodology section. The protein had an estimated molecular weight of 45 kDa, and was predominantly observed in the insoluble fractions of the recombinant *E*. *coli* cells (S2 Fig).

Previous reports have indicated that E. *coli* cells alone produce four major types of fatty acids using type II fatty acid synthase without the influence of fatty acid desaturase enzyme. These fatty acids include myristic acid (C14:0), palmitoleic acid (C16:1), palmitic acid (C16:0), and cis-vaccenic acid (C18:1Δ11), which all are constituents of membrane phospholipids except myristic acid. Moreover, lauric acid (C12:0) and 3-hydroxymyristic acid are made up of the small components of polysaccharide found only in the hydrophilic outer membranes [3, 25].

**Fig 5:**
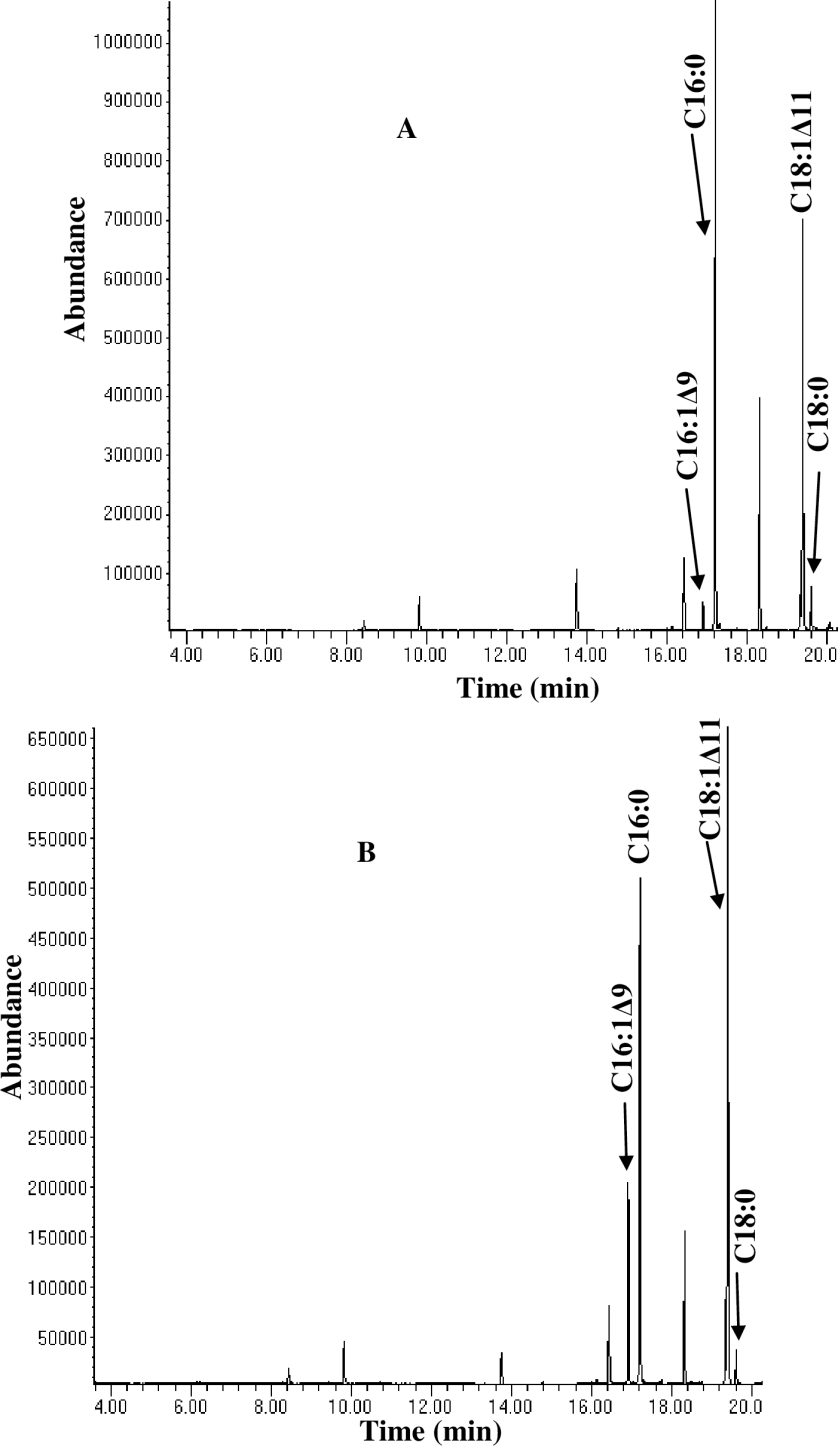
Gas chromatograms of fatty acids identified in *E*. *coli* Transetta (DE3) cells transformed with an empty pET32b vector (A) or cells expressing the recombinant pET32A3DES construct (B). The peaks at the retention time of 9.82, 13.75 and 16.44 min were identified as lauric, myristic, and 3-hydroxymyristic acids, respectively, according to MS data.

**Fig 6:**
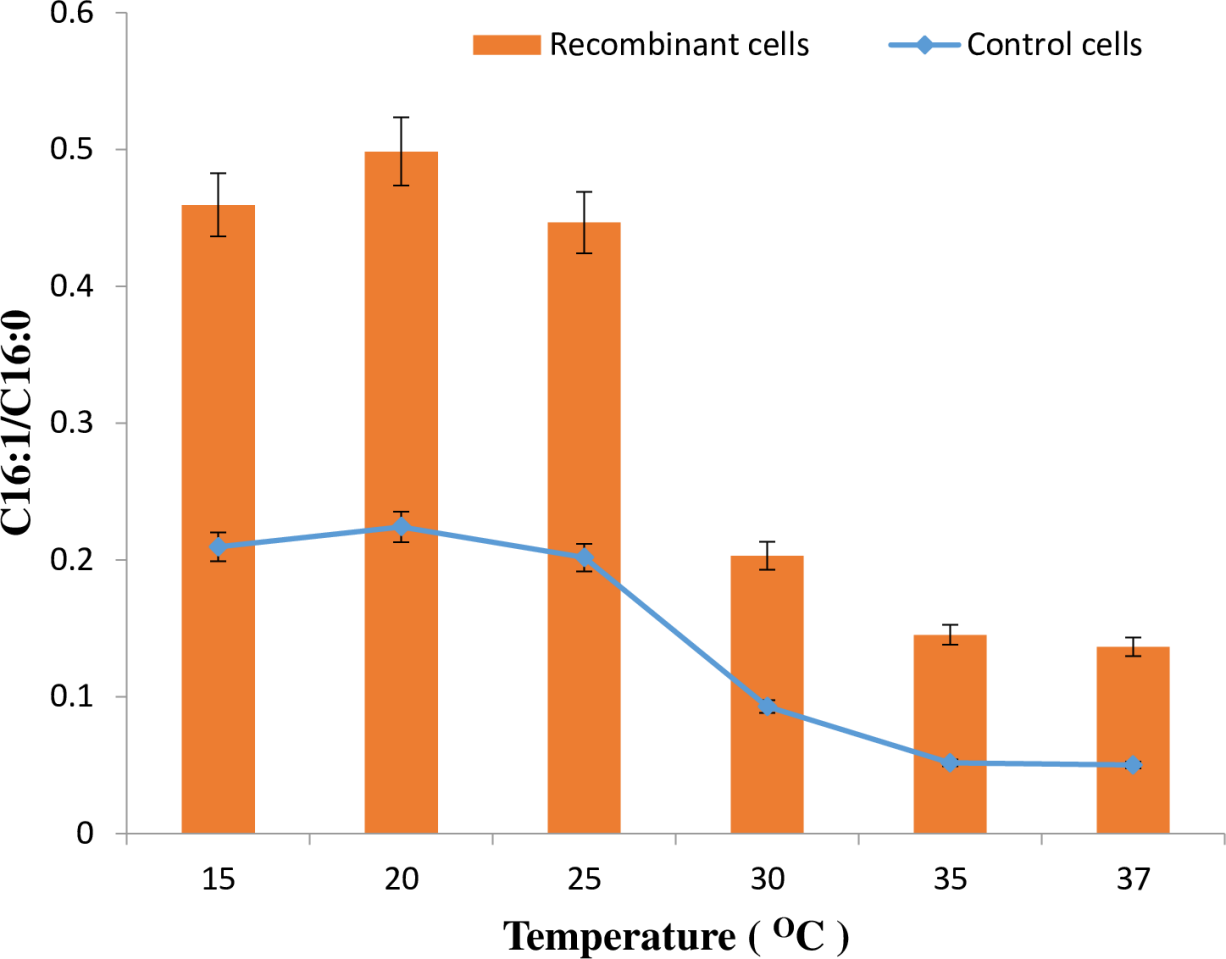
Ratio of palmitoleic to palmitic acid in *E*. *coli* Transetta (DE3) transformed with an empty pE32b (Control cells) or with the pET32A3DES construct (recombinant cells). The cells were collected 12 h after induction at different temperatures. The results are the means of three separate experiments with bars (S. E) and show significant differences between the control and the recombinant cells analysed statistically using one-way ANOVA of variance (P<0.05).

GCMS analysis of cellular fatty acids showed a significant conversion of palmitate to palmitoleate in *E*. *coli* cells during the expression of PA3FAD9 gene (Table 2). The amount of palmitoleate was found to increase with incubation temperatures. However, the highest amount was observed at 15°C, reaching two-fold increase more than the control cells, as confirmed by the GCMS (Fig 5). Moreover, after 12 h of induction at 20°C, the ratio of palmitoleic acid to palmitic acid was approximately 0.50 and 0.22 in *E*. *coli* cells expressing PA3FAD9 and the cells transformed with an empty pET32b vector respectively (Fig 6). The results are in line with the maximum growth temperature of psychrophilic bacteria [26]. The results also demonstrate that PA3FAD9 from *Pseudomonas sp*., A3 codes for a fatty acid desaturase-like protein which was actively expressed in *E*. *coli*.

**Table 2:** Overall fatty acids identified from *E*. *coli* Transetta (DE3) grown at different temperatures.

Temperature (°C)	Fatty Acids							
	C16:1Δ9		C16:0		C18:1Δ11		C18:0	
	a	b	c	d	e	f	g	h
15	10.91	5.64	28.16	40.70	54.56	37.96	6.37	15.70
	±1.33	±0.17	±1.09	±5.66	±2.22	±2.53	±0.01	±0.47
20	10.18	5.70	28.15	38.53	52.34	41.21	9.33	14.56
	±0.74	±0.14	±4.24	±1.70	±3.84	±0.82	±0.36	±0.01
25	9.03	5.20	28.34	40.22	53.02	38.37	9.61	16.21
	±0.52	±0.20	±0.05	±3.70	±1.10	±0.04	±0.34	±0.60
30	4.85	2.79	34.28	41.26	48.41	42.01	12.46	13.94
	±0.31	±0.22	±0.58	±1.90	±480	±4.72	±0.65	±0.80
35	4.98	1.98	43.90	49.06	39.62	35.21	11.50	13.75
	±0.04	±0.63	±1.60	±1.47	±1.50	±3.04	±0.75	±1.50
37	4.17	1.84	42.38	50.83	40.24	31.18	13.21	16.15
	±0.30	±0.16	±2.70	±4.35	±1.54	±2.22	±0.22	±0.30

The overall fatty acids identified in *E*. *coli* cells transformed with an empty pET32b vector or expressing the pET32A3DES construct. The *E*. *coli* cells were cultured at 37°C under reciprocal shaking of 200 rpm until the O.D was approximately 0.5. The culture was induced with 0.1 mM IPTG and shifted the incubation to different temperatures for 12 h. Fatty acid methyl esters were prepared and analysed using GCMS. The major fatty acids identified from both *E*. *coli* cells are C16:1Δ9 (a-b), C16:0 (c-d), C18:1Δ11(e-f) and C18:0 (g-h). The letters a, c, e, g and b, d, f, h represent fatty acids identified from the recombinant and the control cells of *E*. *coli* cells, respectively. Results are means ± S.D of three independent experiments. The amount of palmitoleic acid (C16:1Δ9) produced by the recombinant cells (a) was significantly higher than the control cells (b) throughout the incubation temperatures analysed statistically using one-way ANOVA (p<0.05).

**Fig 7:**
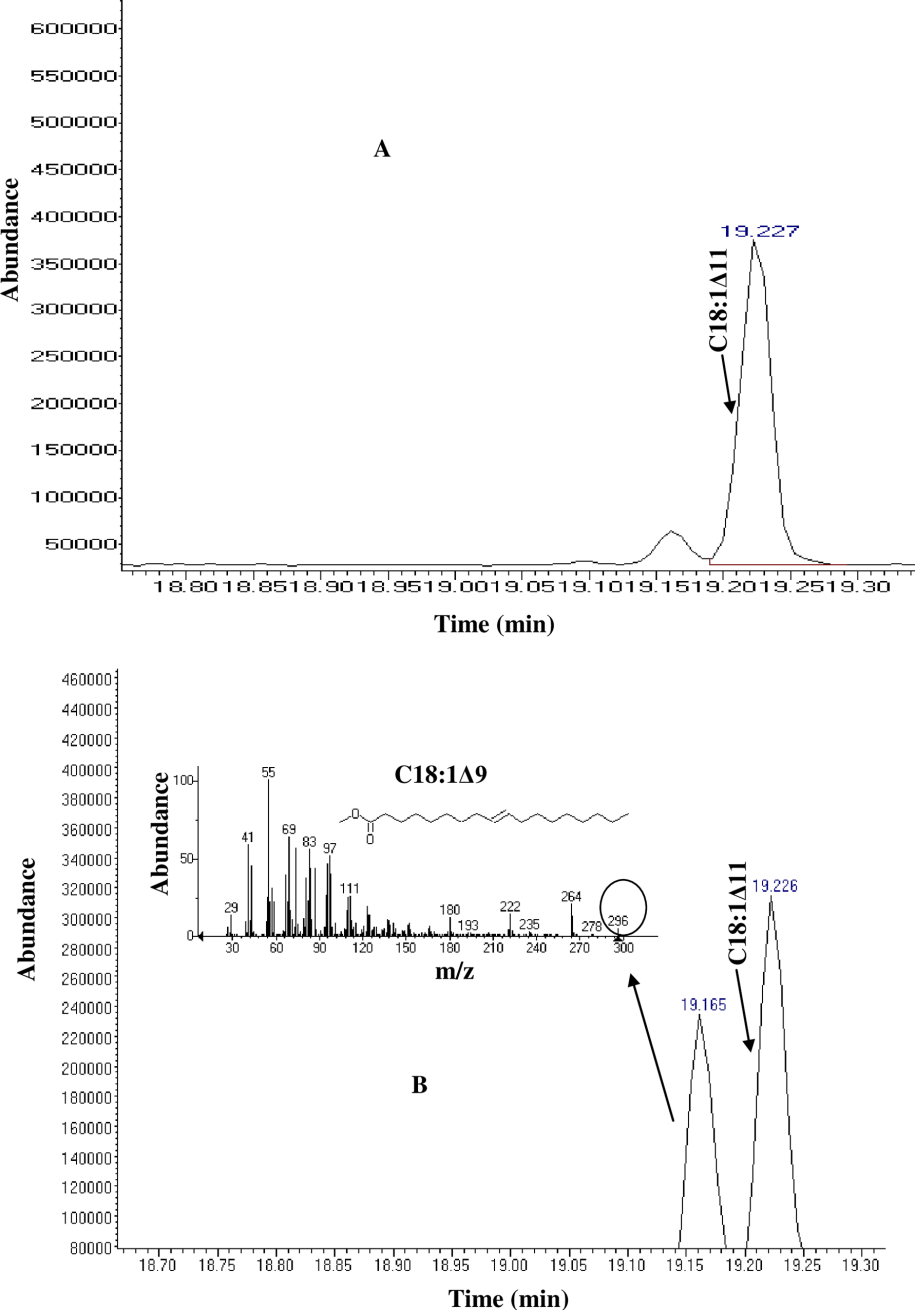
Gas chromatograms of fatty acids identified in *E*. *coli* Transetta (DE3) cells transformed with an empty pET32b vector (A) or expressing pET32A3DES construct (B) grown with 0.4 mM stearic acid. A peak corresponding to oleic acid is shown at the retention time of 19.17 (min) according to MS data.

To confirm the activity of *Pseudomonas sp*., A3 Δ9- fatty acid desaturase, *E*. *coli* cells lacking stearic acid as a substrate for Δ9- fatty acid desaturase [1–2] were grown in Luria Bertani medium supplemented with 0.4 mM stearic acid at 15°C. Their total cellular fatty acids were then analysed (Table 3). This analysis confirmed the ability of *Pseudomonas sp*., A3 Δ9- fatty acid desaturase to convert the exogenous stearic acid incorporated into the *E*. *coli* membrane to produce oleic acid which amounts to about 5%. This was reflected on the GC chromatograms (S3 Fig) as analysed from the *E*. *coli* cells expressing PA3FAD9 gene. Although the newly produced peak that corresponds to oleic acid was not well separated from the cis-vaccenic acid, further analysis showed two separate peaks at the retention times of 19.17 min (Oleic acid) and 19.23 min (cis-vaccenic acid) (Fig 7).

**Table 3:** Fatty acids composition of *E*. *coli* Transetta (DE3) cultured with 0.4 mM stearic acid.

E. coli		Fatty acids				
		C16:1Δ9	C16:0	C18:1Δ9	C18:Δ11	C18:0
**Recombinant cells**		3.15±0.23	3.92±0.11	4.62±0.47	8.68±0.27	80.39±0.10
**Control cells**		4.50 ±0.51	4.60±0.90	ND	10.50±0.85	76.40±0.87

Fatty acids composition of *E*. *coli* Transetta (DE3) transformed with pET32A3DES construct (recombinant cells) or an empty pET32b vector (control cells) were collected after 12 h incubation with 0.4 mM stearic acid and identified using MS library. Results are means ±S.D of three independent experiments with significant production of oleic acid in the recombinant cells. ND: Not detected.

## Discussion

Desaturases and elongases are enzymes that show great potential in the field of biotechnology, especially in the biosynthesis of polyunsaturated fatty acids [3, 27]. Isolation, cloning, and expression of genes coding for these enzymes may boost unsaturated fatty acids production, especially the so-called essential polyunsaturated fatty acids required by the body. This is particularly the case in the event of cloning Δ9-desaturase gene with a gene coding another desaturase/elongase gene and expressed them into a cell capable of producing unsaturated fatty acids through recombinant DNA technology. The Δ9-desaturases catalyse the introduction of the first double bond at the C9 position of palmitate and stearate acids to produce palmitoleic and oleic acids, respectively, which are both initial ingredients required in the synthesis of most polyunsaturated fatty acids [28]. Among the three classes of Δ9- desaturases, only soluble Δ9-acyl carrier protein desaturases have been studied in detail. The rest classes of membrane-bound desaturases could serve as alternative means of enhancing polyunsaturated fatty acids production. There are currently many reports on membrane-bound desaturases already isolated from both prokaryotes and eukaryotic organisms such as cyanobacteria [29–30], *Psychrobacter urativorans* [3], *Pseudoalteromonas* sp. MLY15 [1], fungi [10, 31], higher plants [32], fish [33], and mammals [11]. However, reports on bacterial Δ9- desaturases are still limited compared to higher organisms. In this report, we have successfully cloned and expressed a new gene from *Pseudomonas sp*.A3 that codes for a Δ9- desaturase in *E*. *coli* for the first time to the best of our knowledge. GCMS analysis of the recombinant cells has shown that the gene was actively expressed and that its product was able to catalyse the desaturation of cellular fatty acids of the recombinant cells of *E*. *coli* to produce palmitoleic acid under the influence of temperature and inducer. The *E*. *coli* itself does not contain a stearic acid substrate for Δ9-fatty acid desaturase [1]. However, the *E*. *coli* cells may absorb an exogenous stearic acid substrate into their membrane lipids and become desaturated by *Pseudomonas sp*., A3 Δ9-fatty acid desaturase producing about 5% oleic acid (Table 3). This is higher than the amount reported from *Pseudoalteromonas* sp. MLY15 Δ9-fatty acid desaturase which converted exogenous stearic acid Oleic acid (3%) [1], most likely due to the enzyme preference for palmitoleic acid. Furthermore, the amount of C18:1Δ11 produced by *E*. *coli* cells grown with 0.4 mM stearic acid has significantly decreased (S3 Fig).

The previous report identified and characterised two aerobic desaturase enzymes known as DesA (PA0286) and DesB (PA4888) from *Pseudomonas aeruginosa*. DesA is a phospholipid Δ9-desaturase enzyme whereas the DesB is an inducible acyl-CoA Δ9-desaturase [34]. In this study, we have successfully cloned and expressed an active aerobic phospholipid Δ9- desaturase from an Antarctic *Pseudomonas sp*.A3. Further research needs to be carried out to obtain a soluble phospholipid Δ9- desaturase for *in vitro* analysis and structural studies, particularly by switching to eukaryotic expression systems.

## Materials and Methods

### Bacterial Strains

*Pseudomonas sp*., A3 (GenBank accession no: KR821141) was obtained from Enzyme and Microbial Technology Research Centre, Faculty of Biotechnology and Biomolecular Sciences, University Putra Malaysia. The strain of *E*. *coli* TOP10 (ThermoFisher scientific, USA) was used throughout the routine cloning and plasmid propagation. The *E*. *coli* Transetta (DE3) (TransGen Biotech Co. Ltd, Beijing) enhances proteins expression in the prokaryotic systems and was used for the heterologous expression of desaturase from *Pseudomonas sp*., A3. Storage and cultivation of all strains were strictly based on suppliers’ recommendations.

### Fatty acids Profile of wild-type *Pseudomonas sp*. A3 in relation to temperature changes

*Pseudomonas sp*., A3 was initially grown on two separate nutrient agar plates (Liofilchem, Italy) at 4 and 15°C for 96 h. Subsequently, the bacterium was grown in the same growth medium at 4 and 15°C for 48 h and then changed the former to 15°C and the latter to 4°C for an additional 48 h. The *Pseudomonas sp*.A3 was analysed for fatty acids according to MIDI protocol (Microbial Identification system, Microbial ID Inc., Newark, DE, USA). Approximately 40 mg of bacterial cells was suspended in 1 ml of NaOH (15% w/v) in Methanol/ water (1:1 v/v) and saponified to prepare FAMEs. The FAMEs were extracted using hexane (SupraSolv^®^, Germany) in tertbutyl-methyl ether (1:1, v/v) (Sigma, Aldrich). 1μL of the FAMEs was analysed using QP 2010 GCMS (Shimadzu, Japan) equipped with 30-m HP-5 column (0.25 mm x 0.25 μm) with helium as a carrier gas and a linear velocity of 30 cm^**-**s^. The column temperature was set at 100°C for 5 minutes and later raised to 160°C for 13 minutes with ramping at 20°C per minute. The heating temperature was increased to 250°C with ramping at 10°C per minute and finally held at 250°C for 27 minutes.

### Cloning of desaturase gene

Genomic DNA of *Pseudomonas sp*., A3 was isolated using DNeasy® Blood and Tissue kits (Qiagen, USA) according to manufacturers’ recommendations. Desaturase genes of seven (7) *Pseudomonas species* were obtained from NCBI (http://www.ncbi.nlm.nih.gov) and used for degenerate primers design. These species included *Pseudomonas entomophila* l48 (Accession no: YP_605982.1); *Pseudomonas mendocia* ymp (Accession no: YP_001189787.1); *Pseudomonas stutzeri* A 1501 (Accession no: YP_001170607.1); *Pseudomonas aeruginosa* PAO1 (Accession no: NP_248977.1); *Pseudomonas denitrificans* ATCC (Accession no: YP_007660177.1); *Pseudomonas poae* (Accession no: YP_007399951.1); and *Pseudomonas putida* w619 (Accession no: YP_001751840.1).

The primers were named D1forward (CACCGYAAGCAYCAYGCCAARTGCGARACC) and D2 reverse (NGGRTAGGTGTGRTGRTTGTTRTGCAGYTC) and were used to perform PCR on a thermocycler which was incubated at 94°C for 4 minutes, followed by 35 cycles of 94°C for 1 minute, 60°C for 30 seconds, 74°C for 1 minute and 74°C for 10 minutes with a final holding at 4°C. The amplified fragment demonstrated a high degree of identity to a certain region of *Pseudomonas fluorescence* (Gene accession no: CP010896).

Subsequently, the primers FAD1 (ATGTGGTACAACGGTTTTCTTGATCTGTC) and FAD2 (TCACGCTGCTGTAGGACGCAACGAGTAAG) were derived from the flanking regions of desaturase gene of the P. *fluorescence* and used to isolate the full-length desaturase gene from *Pseudomonas sp*., A3 by performing another round of PCR using the same conditions described above except the annealing temperature (50°C). All gene fragments obtained during the cloning were purified by gel extraction (Qiagen, Germany) and sequenced directly before or after ligation into pGEM ®- T Easy vector (Promega, Germany).

### Sequence Analysis

The entire sequence analysis was performed using a BLAST search programme (http://www.ncbi.nlh.nih.gov/blast). Multiple sequence alignment of putative Δ9-desaturases was carried out using clustal W 3.2 [35]. Analysis of hydrophobic amino acids distribution was carried out using Kyte-Doo-little hydropathy scale with a window size of 19 (http://www.expasy.ch/cgi-bin/protscale.p1) [21]. Transmembrane (TM) regions of the protein were predicted with TMHMM (transmembrane Hidden Markov Model) (http://www.cbs.dtu.dk/services/TMHMM) [36]. Signal sequence analysis was performed using a signal peptide prediction server (htpp://www.cbs.dtu.dk/services/Signal1P-3.0) [37].

### Preparation of Plasmid construct for Gene expression in *E*. *coli*

The complete gene coding putative desaturase from *Pseudomonas sp*., A3 was amplified by PCR using primers F (AGTCGGTACCATGTGGTACAACGGT), and R (ATGCAAGCTTGTCACGCTGCTGT). The underlined sequences stand for restriction endonuclease sites of KpnI and HindIII in the forward and reverse primers respectively. The PCR product was purified, digested with KpnI and HindIII restriction enzymes and ligated into a pET32b expression vector (Novagen, Germany). Successfully transformed colonies were screened on LB agar plates containing ampicillin (100 μg/ml) and Chloramphenicol (34 μg/ml). The recombinant plasmid was designated as pET32A3DES and confirmed by PCR, restriction enzymes digestion, and sequencing.

### Expression of *Pseudomonas sp*., A3 Δ9-fatty acid desaturase in *E*. *coli*

A single colony of *E*. *coli* Transetta (DE3) Transformed with pET32A3DES was propagated in 10 ml of LB broth containing ampicillin (100 μg/ml) and chloramphenicol (34 μg/ml) at 37°C for 16 hrs. An aliquot of the overnight broth culture was used to inoculate 200 mL of fresh LB broth supplemented with the same antibiotics and incubated until the OD600 was approximately 0.5–0.6. At this point, IPTG was added to a final concentration of 0.1 mM and changed the incubation to different temperatures (15, 20, 25, 30, 35 and 37°C). The cells were harvested by centrifugation at 5000 xg, 4°C for 15 minutes. The pellets were re-suspended in 10 mL MOPS-NaOH buffer (50 mM, pH 7.5) and preserved at -80°C until analysis. *E*. *coli* Transetta (DE3) transformed with an empty pET32b vector was given the same treatment conditions to serve as negative control.

### SDS-PAGE Analysis of desaturase

The frozen cells were thawed on ice at room temperature and re-suspended in 10 mL MOPS-NaOH buffer (50 mM, pH 7.5) containing 10 mM MgCl_2_, 1 mM PMSF and lysed by sonication. The lysate was separated by centrifugation at 10,000 xg for 10 minutes at 4°C. Both soluble and insoluble fractions were assayed for protein expression by SDS-PAGE on 12% gel and stained by coomassie staining [38].

### Extraction and Analysis of total cellular fatty acids

Total cellular fatty acids were extracted in four steps according to the MIDI protocol of Microbial Identification system, Microbial ID Inc. (Newark, DE, USA). Approximately 40 mg of wet cells were measured from *E*. *coli* Transetta (DE3) transformed with pET32A3DES construct or an empty pET32b vector and re-suspended in 1 mL of solution 1 (45 g sodium hydroxide, 150 ml methanol, and 150 ml distilled water). The suspension was vortex mixed and directly saponified at 100°C for 30 minutes. The resultant solution was allowed to cool and added 2 mL of solution 2 (325 ml certified 6.0 N hydrochloric acid and 275 ml methyl alcohol), vortex mixed and boiled at 80°C for 10 minutes. The solution was allowed to cool and added 3 mL of solution 3 (200 ml hexane and 200 ml methyl tert-butyl ether), vortex mixed for 10 minutes and removed the inorganic phase. The organic phase was mixed with 3 mL of solution 4 (10.8 g sodium hydroxide in 900 ml distilled water) and vortex mixed for 5 minutes as a final wash. About 1/3 of the upper portion containing the fatty methyl esters were placed into a GC vial for analysis.

The fatty acid methyl esters were separated and analysed via gas chromatography-mass spectrometry via GCMS, QP2010 (Shimadzu, Japan). The GC column used was 30-m HP5 column (internal diameter 0.25 mm, film thickness 0.25 μm) and helium was used as a carrier gas with a linear velocity of 30 m/s. The column conditions are the same as mentioned above (fatty acid profile of wild-type *Pseudomonas sp*., A3).

### Statistical Analysis

The data analysis was performed using one-way ANOVA of variance which showed significant difference at P<0.05 [39–40].

## Supporting Information

S1 Fig PCR product of desaturase gene of *Pseudomonas sp*. A3 analysed on 1% agarose gel. M: 1 kb DNA ladder (250–10,000 bp, Thermo Scientific), Lane 1: PCR product. The arrowhead indicates an estimated size of 1, 200 bp.
(TIF)

S2 Fig SDS-PAGE analysis of *Pseudomonas sp*. A3 Δ9-fatty acid desaturase on 12% gel. The E *coli* cells transformed with an empty vector (control) or pET32A3DES construct were grown at 37°C until the OD was approximately 0.5. The culture was induced with 0.1 mM IPTG and grown at 15°C for 12 h. M (unstained protein maker), 1–2 (soluble and insoluble fractions of control cells), 3–4 (soluble and insoluble fractions of uninduced cells), 5–6 (soluble and insoluble fractions of recombinant cells). The arrowhead shows the overexpressed Δ9-fatty acid desaturase protein with an approximate molecular weight of 50 kDa.
(TIF)

S3 Fig Gas chromatograms of fatty acids identified in *E*. *coli* Transetta (DE3) cells transformed with an empty pET32b vector (a) or cells expressing the pET32A3DES construct (b) grown with 0.4 mM stearic acid. A peak corresponding to Oleic acid was identified at the retention time of 19.17 min based on the MS data.
(TIF)
