# Supplementary figures and images for: Molecular Cloning and Functional Expression of a Δ9- Fatty Acid Desaturase from an Antarctic Pseudomonas sp. A3
Source: PLoS One. 2016 Aug 5;11(8):e0160681. doi: 10.1371/journal.pone.0160681 (PMC4975390; doi:10.1371/journal.pone.0160681)

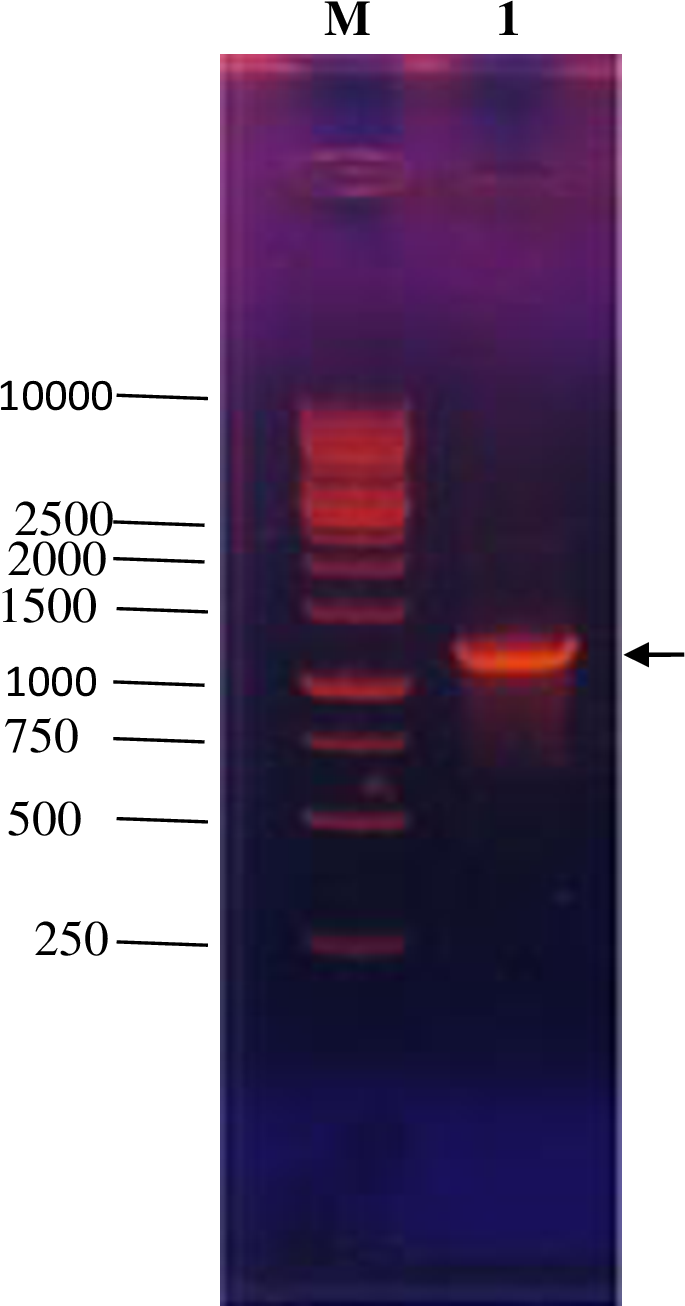

Supplement: S1 Fig — M: 1 kb DNA ladder (250–10,000 bp, Thermo Scientific), Lane 1: PCR product. The arrowhead indicates an estimated size of 1, 200 bp. (TIF) [file pone.0160681.s001.tif]

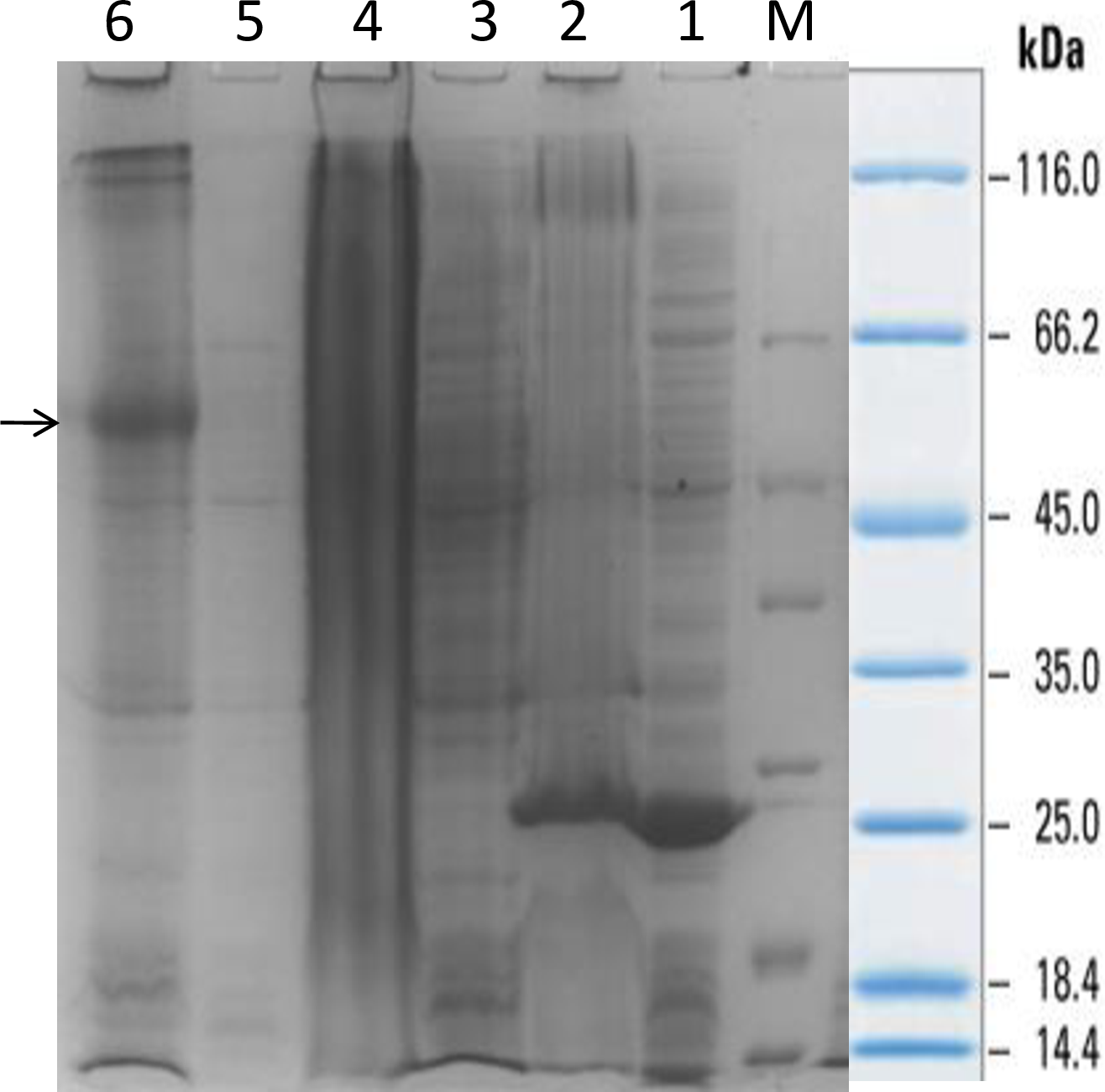

Supplement: S2 Fig — The E coli cells transformed with an empty vector (control) or pET32A3DES construct were grown at 37°C until the OD was approximately 0.5. The culture was induced with 0.1 mM IPTG and grown at 15°C for 12 h. M (unstained protein maker), 1–2 (soluble and insoluble fractions of control cells), 3–4 (soluble and insoluble fractions of uninduced cells), 5–6 (soluble and insoluble fractions of recombinant cells). The arrowhead shows the overexpressed Δ9-fatty acid desaturase protein with an approximate molecular weight of 50 kDa. (TIF) [file pone.0160681.s002.tif]

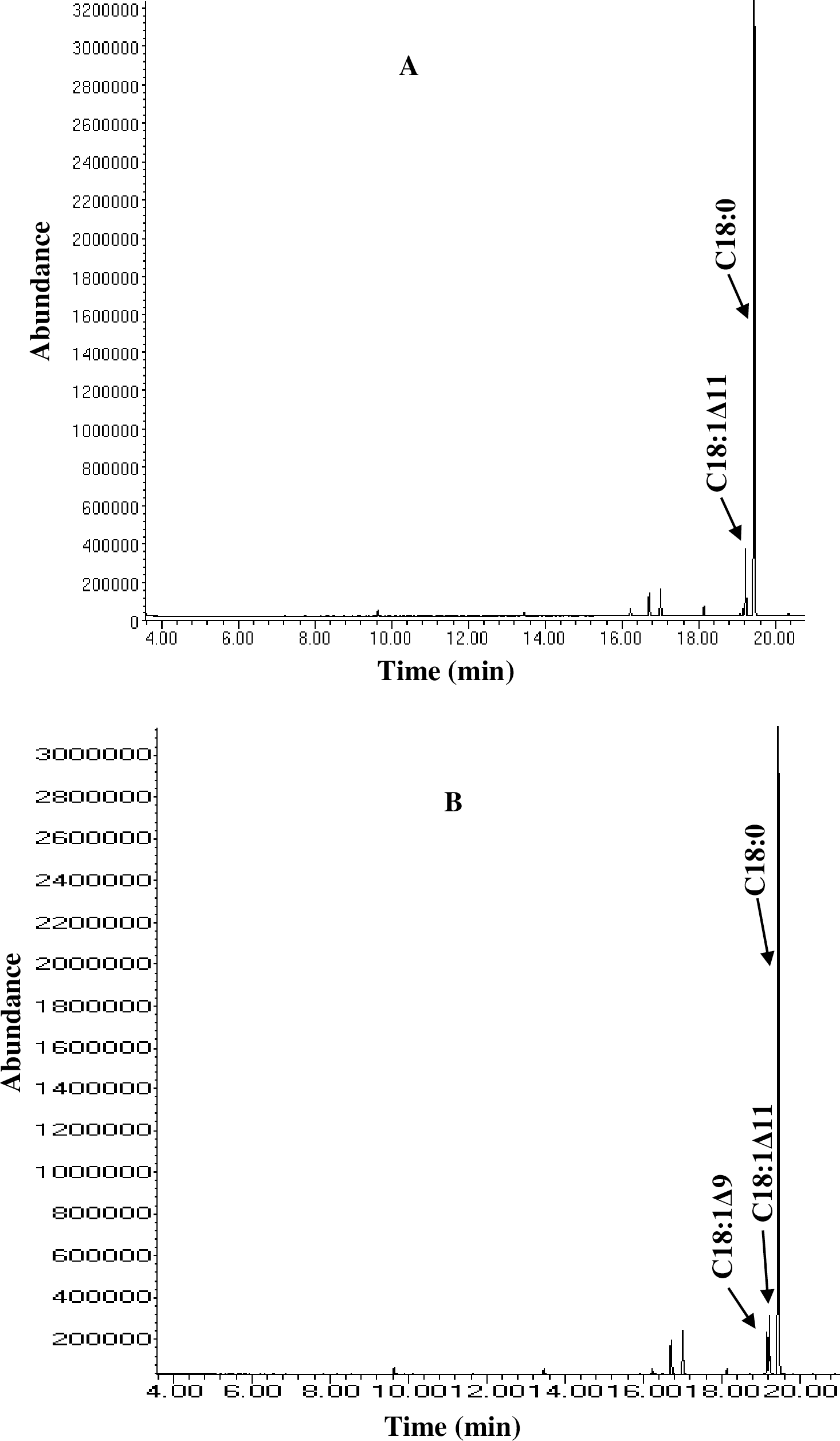

Supplement: S3 Fig — Gas chromatograms of fatty acids identified in E. coli Transetta (DE3) cells transformed with an empty pET32b vector (a) or cells expressing the pET32A3DES construct (b) grown with 0.4 mM stearic acid. A peak corresponding to Oleic acid was identified at the retention time of 19.17 min based on the MS data. (TIF) [file pone.0160681.s003.tif]
